# Knowledge creation in IT projects to accelerate digital innovation: two decade systematic literature review

**DOI:** 10.12688/f1000research.70646.3

**Published:** 2021-12-02

**Authors:** Tung Soon Seng, Magiswary Dorasamy, Ruzanna Razak, Maniam Kaliannan, Murali Sambasivan

**Affiliations:** 1 Multimedia University, Cyberjaya, Malaysia; 2 Alibaba Group Malaysia Sdn Bhd, Kuala Lumpur, 59200, Malaysia; 3 University of Nottingham Malaysia, Semenyih, Malaysia; 4 Thiagarajar School of Management, Madurai, India

**Keywords:** Knowledge Creation, Digital Innovation, Digital Economy, Systematic Literature Review, IT Projects, Information Technology, Transactive Memory System, Trust

## Abstract

The interactivity and ubiquity of digital technologies are exerting a significant impact on the knowledge creation in information technology (KC-IT) projects. According to the literature, the critical relevance of KC-IT is highly associated with digital innovation (DI) for organisational success. However, DI is not yet a fully-fledged research subject but is an evolving corpus of theory and practise that draws from a variety of social science fields. Given the preceding setting, this study explores the interaction of KC-IT with DI. This work provides a systemic literature review (SLR) to examine the literature in KC-IT and its connection to DI. A SLR of 527 papers from 2001 to 2021 was performed across six online databases. The review encompasses quantitative and qualitative studies on KC-IT factors, processes and methods. Three major gaps were found in the SLR. Firstly, only 57 (0.23%) papers were found to examine the association between KC and IT projects. These works were analysed for theories, type of papers, KC-IT factors, processes and methods. Secondly, the convergence reviews indicate that scarce research has examined TMS and trust in KC-IT as factors. Thirdly, only 0.02% (5) core papers appeared in the search relevant to KC in IT projects to accelerate DI. The majority of the papers examined were not linked to DI. A significant gap also exists in these areas. These findings warrant the attention of the research community.

## Introduction

Knowledge is an important asset for promoting organisational change in the 21st century. ^1^ Knowledge comprises a complicated blend of individual experiences, beliefs, relevant information and personal perspective. ^2^ Moreover, knowledge is a driver of worldwide competitiveness in Industry 4.0 (I4.0). Knowledge helps businesses integrate machinery and processes, as complemented by cutting-edge technology. ^3^

Knowledge creation (KC) is an on-going process to acquire new context, views and knowledge and thus transcends the limits of the old to a new self. ^4^ In this study, the theory of organisational knowledge creation (TOKC) from Nonaka and Takeuchi was adopted as the primary theoretical base given its prevalence as the most significant theoretical model in KC studies. ^5^^,^^6^ TOKC explained the organisational KC process through the four modes of conversion including Socialisation, Externalisation, Combination and Internalisation (SECI) of the concepts and embodying knowledge to create product value. ^4^ The Current KC paradigm has shifted to encompass wider areas such as energy, education and high technology. ^7^ A New KC model integrates the SECI process with grey knowledge (half tacit and half explicit knowledge) in high technology projects. The model promotes time as a new dimension in cross-cultural IT industries. ^8^

KC-IT refers to transfer of expertise and knowledge generated at different time in IT project. ^9^ KC-IT does not limited to create documentations but it involves social media that overcomes the limitations in the traditional KC activities. ^10^^,^^11^ For example, project team members create their project knowledge and expertise via social media platform.

IT project interactivity and pervasiveness are shifting the conversation around the value of KC and digital innovation (DI) for organisational performance. ^12^ KC provides valuable and productive outputs to enhance IT projects, it has become a source of global competitiveness in I4.0. DI refers to the application of emerging technologies in a broad variety of innovation. ^13^ Organisations in the digital economy require digital technology to support business innovation. IT project workers today need new skills because they perform in dynamic environments that frequently require new abilities. In this context, DI is essential for general business process and market offerings as technology evolves. ^14^ IT project requires DI to upgrade old processes, leverage emerging technology, build new service channels, and execute new business models.

From the individual perspective, people may benefit from a transactive memory system (TMS) as it enables KC to generate expert knowledge within a community or organisation. ^15^ Past KC literature stressed trust as an important feature for the externalisation of tacit knowledge. ^4^ However, hardly any empirical evidence on TMS and trust on KC was provided. Given the above context, this work seeks to answer the call from Pagona *et al*. ^16^ and Holmström ^17^ to dive further into the intricacies of DI. We aim to highlight the research gaps in KC in IT project research and it is an important component for DI. A total of 57 papers were found relevant to this study.

This study’s research questions are as follows:

1. Is there a research gap in KC-IT in connection to DI?
2. Is there a research gap in TMS and trust affecting KC-IT?
3. What is the current view of KC-IT literature in terms of the KC process, method and factor?
4. What are the underlying theories used by the literature?

The research objectives of this work are as follows:

1. To identify research gaps in KC-IT linking to DI.
2. To evaluate TMS and trust as a possible element for KC-IT
3. To understand the current view of the KC-IT literature in terms of the KC process, method and factor.
4. To identify the underlying theories used by the literature.

## Review method

This work offers a systematic literature overview to identify research gaps and limitations in KC-IT on DI. Key aspects in the KC-IT toward attaining DI were investigated using TOKC as a theoretical basis. The systematic literature review was conducted according to the five stages proposed by Tranfield *et al*. ^18^:

a. Planning the review;
b. Identifying and evaluating studies;
c. Extracting and synthesising data;
d. Reporting descriptive findings; and
e. Utilising the findings to inform research and practice.

### Institutional Review Board Statement

Institutional Review Board Statement: Research Ethical Committee (REC) of Multimedia University (EA1382021). The study was conducted according to the guidelines and approved by the Research Ethical Committee (REC) of MULTIMEDIA UNIVERSITY.

### Stage 1: Planning the review

**Figure 1.**
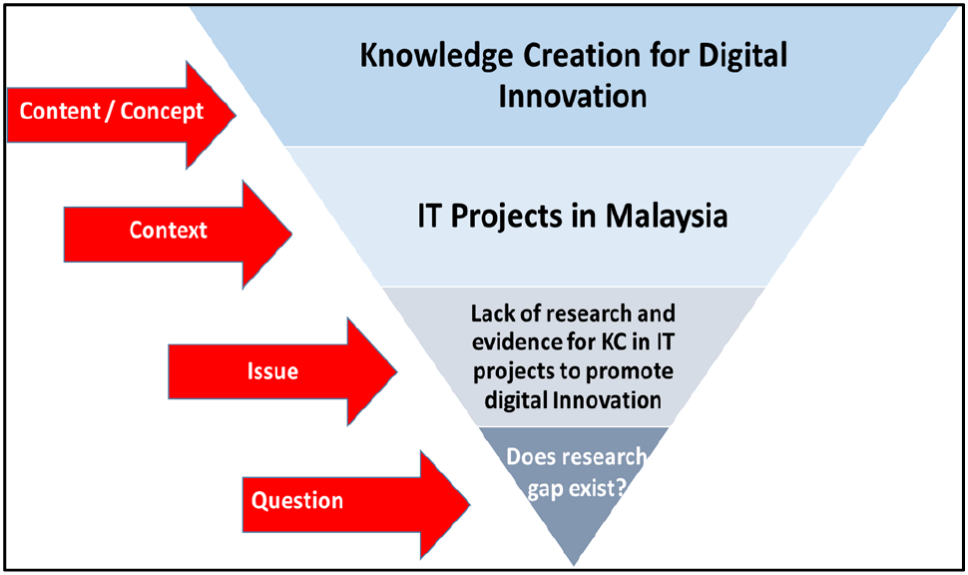
Scope of the review.

This paper provides a comprehensive overview of existing work with emphasis on established and emerging critical factors. TMS and trust as KC factors in IT were investigated. Figure 1 shows this study’s scope.

The strategy for the selection of databases and methods are based on Moher *et al*. ^19^ Methods include searching keywords around terms for KC (the concept) and IT projects (the context) in online databases, including AISeL, IEEE, Emerald, SSCI, Scopus and ProQuest.

### Stage 2: Identifying and evaluating studies

The study’s keywords cover context and content. The search found 24,293 KC papers, but only 527 had keywords for IT projects ( Table 1). Per the criteria, only 57 papers actually addressed KC in IT projects. These papers were classified using Mitchell and Boyle’s ^20^ three major KC dimensions. The KC process refers to the investigations of the measurements or practices performed within KC. The KC factors refers to variables that contribute causally to KC, and the KC method focuses on employing tools or solutions to improve KC.

**Table 1.** Number and percentage of papers on KC.

Detail	No. of papers	Percentage over total KC papers
**Total papers on KC related to IT projects**	527	2.1%
**Selected papers (KC+IT, DI)**	57	0.23%
**Total papers on KC**	24,293	

*Inclusion and exclusion criteria*

**Figure 2.**
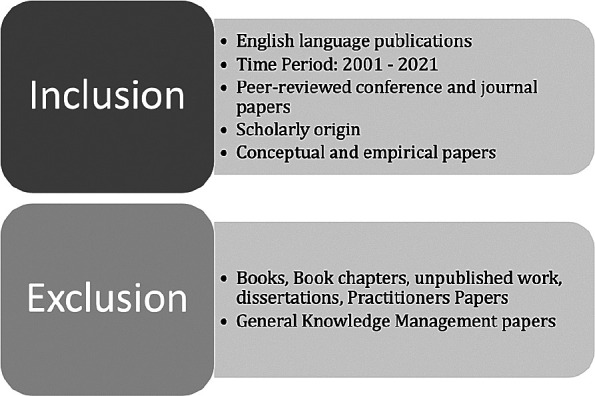
Inclusion and exclusion criteria.

The inclusion and exclusion criteria for the paper search are presented in Figure 2.

*Keywords*

We focused on two main research areas: (1) KC, (2) IT projects, and (3) DI. For the first area, we included terms such as ‘knowledge creation’ and ‘KC’ (abbreviations). The next key terms used were ‘project’, ‘IT project’, ‘IT projects’ and ‘digital innovation’. Each of these keywords was searched with the keyword ‘Knowledge creation’ individually. The search was subsequently extended by adding more keywords. Table 2 presents the keyword sets used for this research.

**Table 2.** Keyword combination sets.

Individual keywords category				Combination sets		
1	2	3	4	5	6	7
Knowledge Creation	Project	IT Project	Digital	Knowledge Creation + Project	Knowledge Citation + IT Project	Knowledge Creation + IT Project + Digital Innovation
KC or Knowledge Creation	Project or Projects or Project Management	IT Project or IT Projects or Information Technology Project	Digital Innovation or Digital Innovations	Knowledge Creation and Project or Projects	Knowledge Creation and IT Project or IT Projects or Information Technology Project or Information Technology Projects	Knowledge Creation and IT Project or IT Projects or Information Technology Project or Information Technology Projects and Digital Innovation

*Search strategy*

We sifted through papers that discussed KC in IT projects for DI. Our strategy was to identify papers through major online databases. We searched six online databases that encompass a vast range of KC as well as IT project-related research and are popular databases for social science study.

1. Association of Information Systems Electronic Library (AISeL)
2. Emerald
3. ProQuest
4. Scopus
5. IEEE
6. Science Direct

**Figure 3.**
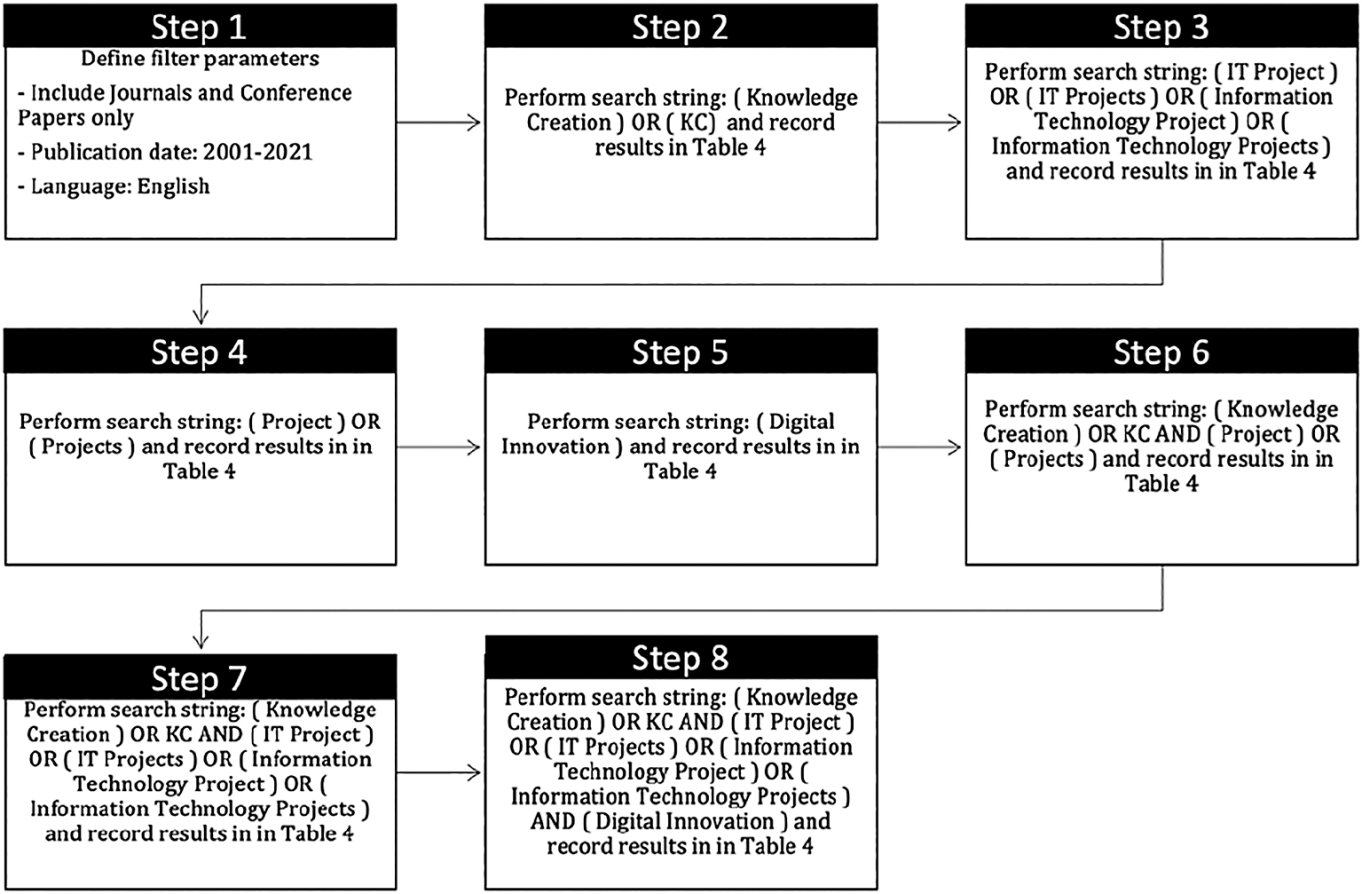
Detail of search strategy.

A detailed of search strategy is presented in Figure 3.

### Stage 3: Extracting and synthesising data

**Figure 4.**
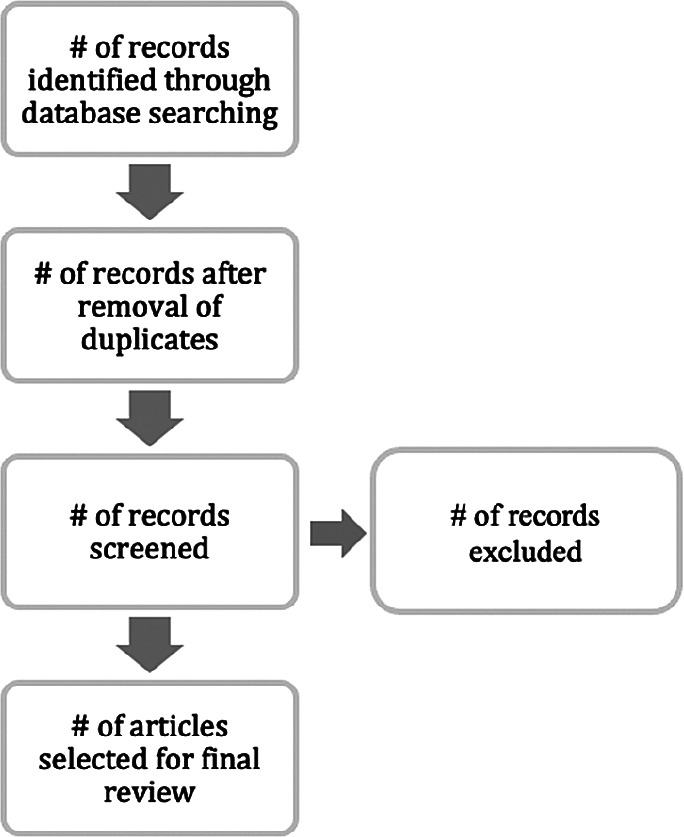
Flow of the extraction process.

We extracted papers from the aforementioned sources on the basis of the following extraction process ( Figure 4).

Figure 4 recaps our basis for selecting papers to review. The extraction process was adopted from Moher *et al*. ^19^ As indicated regarding the main databases and other options that were utilised, only KC papers linked to IT projects and/or DI were selected for further review. The following subsection presents a report of the papers that were relevant according to our selection criteria.

Stages 3, 4 and 5 of Tranfield *et al*. ^18^ will be presented in the form of findings and the discussion.

## Result

Table 3 presents the outcomes from the inclusion conditions and the extraction process mentioned above. A total of 527 papers were identified by referring to the keyword search for KC-IT. 57 papers were found for the keyword search KC-IT-DI which belongs to subset of KC-IT. In this part, we further categorised the papers to indicate their respective types.

**Table 3.** Number of papers by country.

Country	No of papers
Australia	3
Brazil	3
Canada	1
Chile	1
China	3
Czech Republic	1
Denmark	1
Ecuador	1
Finland	1
France	1
Germany	1
Iceland	1
India	2
Iran	4
Italy	1
Japan	2
Malaysia	1
Netherland	2
Nigeria	1
Poland	1
Russia	1
Serbia	1
Slovakia	1
South Africa	3
South Korea	3
Spain	1
Tanzania	1
Thailand	1
Turkey	1
UK	1
US	3
Vietnam	1

**Figure 5.**
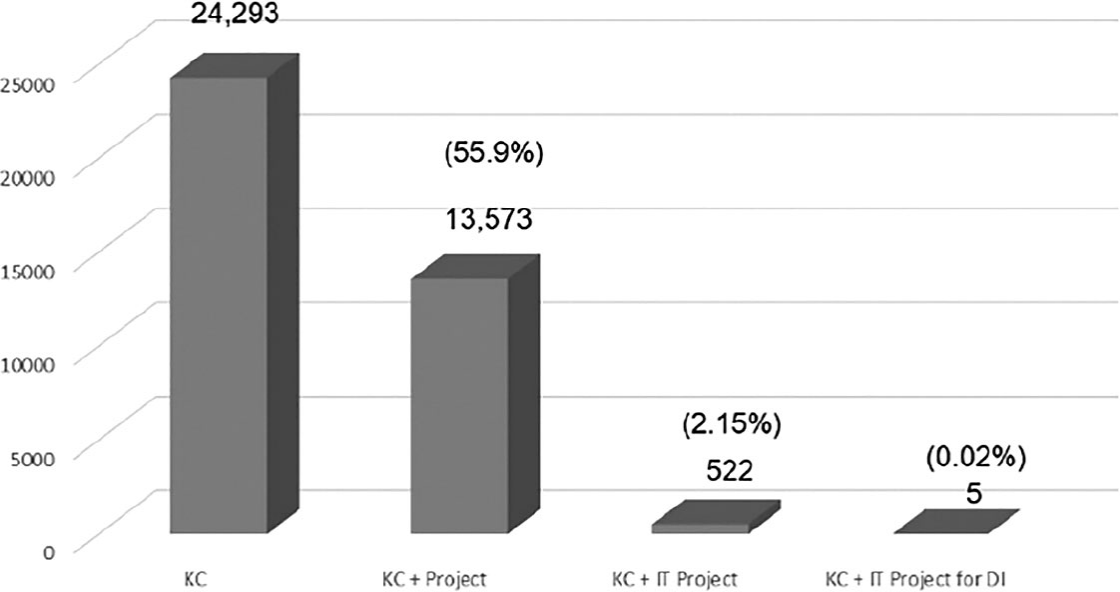
KC papers by categories.

Figure 5 provides a bar chart to highlight the research gap according to the keyword search of KC, KC + Project, KC + IT Project and DI. The KC papers amounted to 24,293. The fractions of the total KC papers can be seen as 55.9% (13,573) on KC in projects, 2.15% (57) on KC in IT projects and 0.02% (5) papers were related to KC in IT project for DI. Figures 5 and 7 were able to meet the study's objective and indicate KC-IT research gaps.

**Figure 6.**
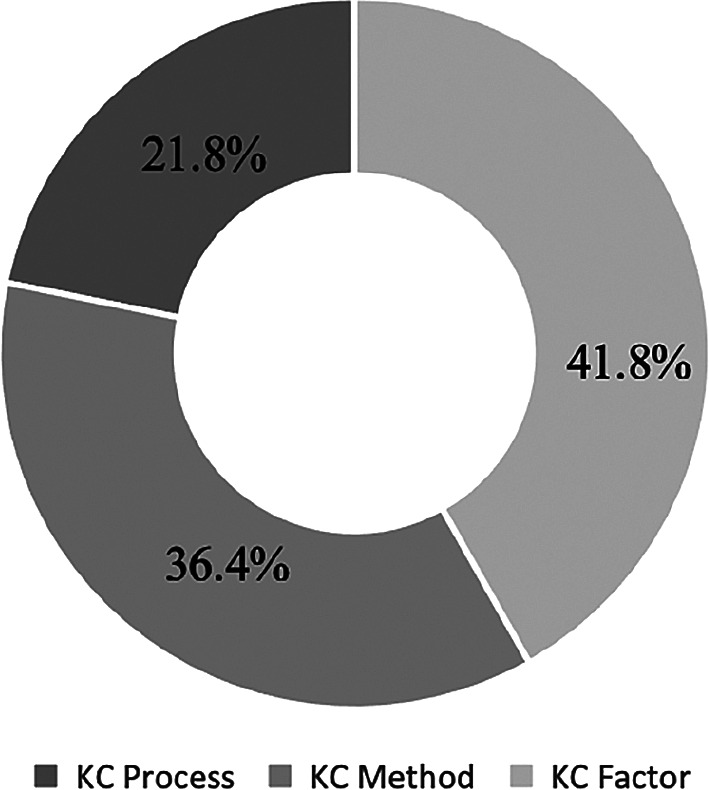
Percentages of KC in IT project papers under three sub categories.

The KC + IT Project papers are divided into three sub categories: KC Process, KC Method and KC Factor. The number of units is indicated in the parentheses, and a pie chart is presented in Figure 6 to reflect the percentages. Figure 6 depicted the objective of the study to understand the current view of the KC-IT literature in terms of sub categories. Figure 4 reveals that 41.8% of the research papers are sub categorised under the KC Factor and 36.4% under the KC Method. Meanwhile, 21.8% papers were related to the KC Process.

**Figure 7.**
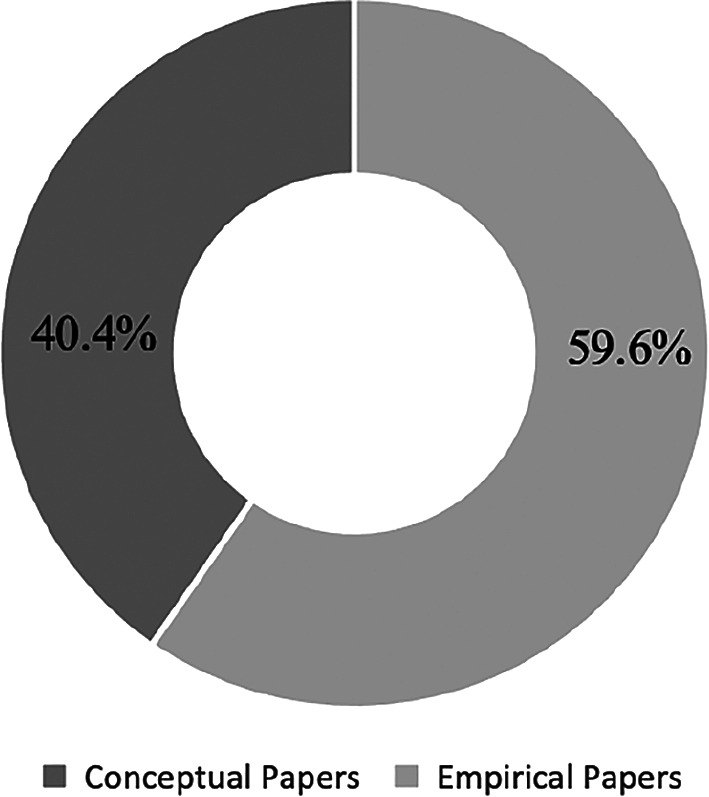
Percentages by paper type.

The papers are divided into two main categories of conceptual and empirical papers. A total of 23 conceptual papers (40.4%) and 34 empirical papers (59.6%) were identified. Conceptual papers lack actual test findings. On the contrary, empirical papers consist of evidence-based research and inputs for testing and findings. Figure 7 presents the percentages of papers by categories.

A total of 50 countries were involved in empirical research ( Table 3). Iran has the highest count of empirical research (4 papers), followed by Australia, Brazil, China, South Africa and United States with 3 papers each.

The complete summary of all the 57 papers is shown in Tables 5 and 6 and according to 3 categories: the KC Method (20 papers), KC Factor (23 papers) and KC Process (12 papers).

## Discussion

### Research gap in KC in IT projects for digital innovation (KC-IT-DI)

Only two papers, written by Ordieres-Meré *et al*. ^21^ and Van den Berg, ^22^ were pertinent to KC in IT projects for DI. The key findings in Table 5 revealed that 0.9% papers are related to KC in IT projects for DI ( Table 5). Although initial search for the keywords linking KC-IT-DI enlisted 57 papers, we found that only 5 papers somehow touch base about KC-IT-DI. We identified these 5 papers by scrutinizing all the 57 papers. However, these 5 papers are conceptual papers. The first paper was written by Ordieres-Meré *et al*. and stated that Industry 4.0 is considered to have a strong association with economic, environmental and social. ^21^ The second paper was written by Van den Berg who developed a paradigm for DI skills encompassing ‘meta-knowledge’ which is the information necessary to drive soft skills. ^22^ The rest of the papers include the work of Park *et al*. who presented novel concepts for organising work. ^23^ Kyakulumbye *et al*. found that relevance and usability are crucial for evaluating systems. ^24^ Shimamoto analysed the strategy for Japanese chemical industry R&D from 1980 to 2010. ^25^ Theoretically, we found that Kyakulumbye *et al.*, Shimamoto and Park *et al.* papers are still far from our search to find literatures that links KC to DI for IT projects. The body of knowledge relating to KC to achieve DI for IT Projects is still scant.

### TMS and trust affecting KC-IT-DI

TMS and trust were found to be important factors to KC-IT. However, the key findings in Table 4 shows two journals that identify TMS as positively related to KC. ^26^^,^^27^ Four journals examine the trust relationship with KC but did not associate their frameworks with DI. This situation is a new research gap for us. ^28^^-^^31^ We proposed that this research gap should be filled according to the theoretical framework ( Figure 7).

**Table 4.** Search result. Table 4 shows the details of the search results by keywords and units of analysis.

No.	Online database	Keywords combinations							Unit of analysis
		Knowledge creation or KC	Project or Projects	IT Project or IT Projects or Information Technology Project or Information Technology Projects	Digital Innovation	Knowledge Creation or KC AND Project or Projects	Knowledge Creation or KC AND IT Project or IT Projects or information Technology Project or Information Technology Projects	Knowledge Creation or KC AND IT Project or IT Projects or Information Technology Project or Information Technology Projects AND Digital Innovation	(Selected papers)
							
1	AISeL	2,642	30,562	3.842	1,387	191	191	0	2
2	Emerald	134	4,050	48	38	4,177	180	0	32
3	ProQuest	97	12,004	5	14	3,520	34	5	19
4	Scopus	20,944	1,335,675	3,206	1,069	988	7	0	0
5	IEEE	200	17,592	230	23	4,685	13	0	1
6	ScienceDirect	276	23,917	23,917	50	12	37	0	3
	Total	24,293	1,423,800	31,248	2,581	13,573	522	5	57

**Table 5.** Summary of KC method papers in IT projects. Table 5 shows the details of 20 KC method papers by the theory used, respondent group and key findings.

	Author	Theory used	Respondent group	Method user	Key findings
1	^37^Mir & Rahaman (2003)	Theory of Organizational Knowledge Creation	Organization workers	Inter-team collaboration	Workers’ experiences and opinions are seen as a vital sources of new knowledge by the firm.
2	^38^Kamimaeda, Izumi & Hasida (2007)	Discourse Semantic Authoring	Organization workers	Group discussion	Participants’ knowledge contributions were evaluated primarily on the substance of their arguments rather than the quantity of comments they made.
3	^13^Balestrin, Vargas & Fayard (2008)	Theory of Organizational Knowledge Creation	Firm managers	Firm network	Knowledge creation process can be developed by a network’s inter-relational structure.
4	^39^Ha, Okigbo & Igboaka (2008)	Theory of Organizational Knowledge Creation	Farmers	Broadband internet and computer	Customised information and socialising functions are critical to gaining support in a knowledge creation.
5	^20^Mitchell & Boyle (2010)	Knowledge creation measurement methods	-	-	Three major dimensions of KC classifications: Process, Method and Factor.
6	^40^Wu, Senoo & Magnier-Watanabe (2010)	Theory of Organizational Knowledge Creation	-	-	An ontological shift SECI model was suggested as a tool for diagnosing organisations in knowledge creation.
7	^41^Song, Uhm & Yoon (2011)	Theory of Organizational Knowledge Creation	IT firms manager	Expert review	Discovered new methodical approach of scale development.
8	^42^Zurita & Baloian (2012)	Theory of Organizational Knowledge Creation	Mobile device users	Software application	Geo-referencing software aids in the conversion of tacit into explicit knowledge.
9	^43^Durst, Edvardsson & Bruns (2013)	Theory of Organizational Knowledge Creation	Small and medium enterprise firms	Network activities	To produce knowledge, SMEs employ knowledge sources prioritise friendly enterprises in the same industry.
10	^44^Esterhuizen *et al.* (2013)	Theory of Organizational Knowledge Creation	-	-	Knowledge creation is a critical facilitator in the development of innovation capacity.
11	^45^Suorsa (2015)	Play theory	-	-	The way of being in knowledge creation interaction may be explained by playfulness, which is absolute present in the event and immersion in the dialogue.
12	^46^Brix (2017)	Theory of Organizational Knowledge Creation, Organizational learning theory	IT project members	Inter-team collaboration	A paradigm for organisational learning and knowledge development that is integrative.
13	^47^Elsa & Runar (2018)	Theory of Organizational Knowledge Creation	Small and medium enterprise managers	Open discussion with customers, suppliers, and research institutions	Team expertise and teamwork are crucial components to generates new knowledge.
14	^34^Faccin & Balestrin (2018)	Theory of Organizational Knowledge Creation	Research & Development (R&D) engineers	Collaborative practice	Atheoretical framework to examine variables of collaborative practice in R&D projects.
15	^48^Li, Liu & Zhou (2018)	Theory of Organizational Knowledge Creation	-	-	A new KC model to integrate SECI process with grey knowledge (half tacit and half explicit knowledge) in high technology projects.
16	^49^Salehi *et al.* (2018)	Theory of Organizational Knowledge Creation	Medical practitioners	Conference and clinical unit	Themes for KC included scientific debate, exchanging clinical experiences, attending conferences, and creating interpersonal relationships.
17	^8^Chin *et al.* (2020)	Theory of Organizational Knowledge Creation	-	-	Introduce Polychronic KC to promote time as the new dimension in cross-cultural IT industries.
18	^50^Choi & Gu (2020)	Theory of Organizational Knowledge Creation	Factory managers	Online meeting	Knowledge produced from knowledge providers regardless of physical proximity.
19	^51^Wang & Li (2020)	Evolutionary game theory	Enterprise community	Community of practice	Using an effective competitive mechanism to promote KC.
20	^52^Pokrovskaia *et al.* (2021)	Theory of Organizational Knowledge Creation	Universities	Online course	Online course for students are crossed with digital instruments ensuring the socio-psychological aspects of the learning process.

**Table 6.** Summary of papers on KC factors in IT projects. Table 6 shows the details of 23 KC factor papers by the theory used, respondent group and key findings.

	Author	Theory used	Respondent group	Key findings
1	^53^Miyashita (2003)	Theory of Organizational Knowledge Creation	Manufacturing firm employees	Organizational effectiveness is linked to knowledge creation and information technology.
2	^51^Merx-Chermin & Nijhof (2005)	Innovative organisations	-	-
3	^30^Teerajetgul & Charoenngam (2006)	Theory of Organizational Knowledge Creation	Project teams	IT support significant affects knowledge creation combination and internalization mode. Collaboration has a strong impact on socialization and externalization.
4	^26^Dunaway & Sabherwal (2012)	Transactive Memory System, Knowledge Management Theory, Theory of Organizational Knowledge Creation	Organization workers	Team transactive memory systems improve the knowledge creation process, which has an impact on team performance.
5	^54^Siadat *et al.* (2012)	Social capital theory, Organizational culture theory	Universities	Organizational culture and social capital significantly influenced knowledge creation.
6	^28^Castro & Sánchez (2013)	Theory of Organizational Knowledge Creation, Concept of Ba	-	New types of leadership and contextual factors such as goodwill, trust, cohesion, commitment, ethic of contribution, high care, atmosphere, wise leadership, love and friendship in the knowledge creation and transfer process.
7	^29^Sankowska (2013)	Theory of Organizational Knowledge Creation	Firm employees	There is positive association between organizational trust and knowledge creation.
8	^55^Thang, Quang & Nguyen (2013)	Resource-based view, Theory of Organisational Knowledge Creation	Firm employees	Knowledge creation processes were affected by a combination of leadership, teamwork, corporate culture, and human resource management.
9	^56^Lee, Park & Kim (2014)	Theory of Organizational Knowledge Creation	R&D workers	Organizational identity and human capital of workers had positive effects on their knowledge creation.
10	^57^Begoña Lloria & Peris-Ortiz (2014)	Knowledge Creation Enablers	Firm employees	Knowledge creation enables such as intention, autonomy, redundancy, variety and trust and commitment have a positive and significant relation with knowledge creation.
11	^58^Nair, Ramalingam & Ashvini (2015)	Knowledge Creation Enablers	Automobile industry workers	All four factors expected have positive impact on knowledge creation.
12	^59^Mikhaylov (2016)	Theory of Organizational Knowledge Creation	Universities	Cultural curiosity influences intrinsic motivation to engage in cultural knowledge creation and sharing.
13	^33^Wang, Zhang & Li (2017)	Knowledge-based view	R&D workers	Competence trust has a positive effect on knowledge creation. Goodwill trust has U-shape relationship with knowledge creation.
14	^60^Papa *et al.* (2018)	Theory of Organizational Knowledge Creation	Small and medium enterprise firms	Social media promote knowledge creation through socialization, externalization, and combination.
15	^33^Thani & Mirkamali (2018)	Theory of Organizational Knowledge Creation	Universities	Personal, institutional, and support factors were found to influence knowledge creation.
16	^61^Cauwelier, Ribiere & Bennet (2019)	Team psychological safety	Engineering teams	Team safety and team learning positively impact team knowledge creation.
17	^27^Çetin (2019)	Knowledge creation capability, Transactive memory system	Firm employees	Transactive memory systems have effects on knowledge creation capability.
18	^62^Mohammed, Baig, & Gururajan (2019)	Talent management processes	Universities	There is a direct influence between talent management processes and knowledge creation
19	^63^Stojanović-Aleksić, Nielsen & Bošković (2019)	Resource-based theory, Theory of organizational knowledge	Organization workers	Organic structure and organizational culture has a positive influence on knowledge
20	^64^Goswami & Agrawal (2020)	Theory of Organizational Knowledge Creation	IT companies	Shared goals and hope have a direct impact on knowledge sharing and creation.
21	^65^Tajedini & Tandiseh (2020)	Information culture theory	Universities	Culture of information increase organization’s knowledge creation.
22	^66^Yoon *et al.* (2020)	Systems model of creativity	Public service organization	Creativity and knowledge creation have a positive association.
23	^31^Tootell *et al.* (2021)	Organizational justice theory, Relationship marketing theory	University, industrial workers	Knowledge creation are fostered by shared value and trust.

**Table 7.** Summary of KC factor papers in IT projects with variables. Table 7 shows the details of KC Factors by independent variables, dependent variables and whether the papers mentioned TMS and Trust.

	Author	Independent variable	Dependent variable	Transactive memory system	Trust
1	^53^Miyashita (2003)	Knowledge creation, Information technology	Organization effectiveness, Organization management		
2	^67^Merx-Chermin & Nijhof (2005)	Strategic alignment, structure, climate, systems, leadership	Knowledge creation process, innovation, Learning		
3	^30^Teerajetgul & Charoenngam (2006)	Vision, Incentive, Collaboration, Trust, IT support, Individual competency	Knowledge creation process		
4	^26^Dunaway & Sabherwal (2012)	Transactive Memory System, IT support for KM	Knowledge creation, Knowledge sharing, Knowledge application, Team performance	√	
5	^54^Siadat *et al.* (2012)	Organizational culture, Social capital	Knowledge creation		
6	^28^Castro & Sánchez (Z013)	Goodwill, trust, cohesion, commitment, ethic of contribution, high care, atmosphere, wise leadership, love and friendship.	Knowledge creation		√
7	^29^Sankowska (2013)	Organizational trust	Knowledge transfer, Knowledge creation, innovativeness		√
8	^55^Thang, Quang & Nguyen (2013)	Leadership, teamwork, corporate culture, and human resource management.	knowledge creation		
9	^56^Lee, Park & Kim (2014)	Organizational identity, Mobility direction, Human capital	Knowledge creation		
10	^57^Begoña Lloria & Peris-Ortiz (2014)	Intention, Autonomy, Fluctuation, Redundancy, Requisite Variety, Trust, Commitment, Creative Chaos	Knowledge creation		
11	^58^Nair, Ramalingam & Ashvini (2015)	Organizational communication, Feedback promotion, Policy formulation, Information sharing	Knowledge creation, Organisational performance		
12	^59^Mikhaylov (2016)	Cultural curiosity	Intrinsic motivation, Knowledge creation		
13	^33^Wang, Zhang & Li (2017)	Competence trust, Goodwill trust	Knowledge creation		√
14	^60^Papa *et al.* (2018)	Social media	Knowledge creation process, Innovation		
15	^32^Thani & Mirkamali (2018)	Personal factors, Institutional factors, Support factors	Knowledge creation		
16	^61^Cauwelier, Ribiere & Bennet (2019)	Team safety, Team learning	Knowledge creation		
17	^27^Çetin (2019)	Transactive memory system, Collective mind, innovative culture	Knowledge Creation Capabilities	√	
18	^62^Mohammed, Baig & Gururajan (2019)	Talent retention, development, attraction	Knowledge creation		
19	^63^Stojanović-Aleksić, Nielsen & Bošković (2019)	Organic Structure, Organizational Culture	Knowledge creation, Knowledge sharing		
20	^64^Goswami & Agrawal (2020)	Shared goal, Hope	Knowledge creation, Knowledge sharing		
21	^65^Tajedini & Tandiseh (2020)	Information culture	Knowledge creation		
22	^66^Yoon *et al.* (2020)	Creativity	Knowledge creation		
23	^31^Tootell *et al.* (2021)	Opportunistic behaviour, Trust, Shared value	Knowledge creation		√

**Table 8.** Summary of KC process papers in IT projects. Table 8 shows the details of 12 KC process papers by the theory used, respondent group and key findings.

	Author	Theory used	Respondent group	Key findings
1	^68^Kippenberger (1997)	Theory of Organizational Knowledge Creation	Organization workers	Organizational knowledge creation making accessible and amplifying knowledge developed by people, as well as crystallising and linking it with an organization’s knowledge system.
2	^69^Eliufoo (2008)	Theory of Organizational Knowledge Creation	Construction firms manager	Social characteristics are critical for organisations to improve knowledge.
3	^70^Spraggon & Bodolica (2008)	Theory of Organizational Knowledge Creation	IT firms manager	Discovered virtual socialization mode in IT software firms.
4	^71^Matysiewicz *et al.* (2013)	Theory of Organizational Knowledge Creation	Scientific networks participants	Participants are more engaged, that partnerships are more established, and there are more prospects for publishing and research.
5	^72^Naicker, Govender & Naidoo (2014)	Theory of Organizational Knowledge Creation	Electrical and Electronics engineers	Engineers use socialization and externalization modes of knowledge conversion, but internalization is important in knowledge creation and transfer.
6	^73^Marsina *et al.* (2015)	Theory of Organizational Knowledge Creation	IT firms manager	There is lack of IT adoption in Slovakia enterprises for their project activities.
7	^74^Shongwe (2015)	Organisational learning theory, Learning Organisation, Theory of Organisational Knowledge Creation, Knowledge-integration theory, Communities of practice theory	Software engineers	Engineers can produce knowledge from a variety of sources, including presentations, from the lectures, the Internet, older students, and professional developers.
8	^75^Yao, Han & Li (2015)	Theory of Organizational Knowledge Creation	Aerospace firm managers	Integrate Chinese philosophy I-Ching into dynamics of knowledge creation.
9	^76^Moraes *et al.* (2016)	Theory of Organizational Knowledge Creation	Electrical and Electronics engineers	The new process of group socialization is used to foster a network of internal connections in order to generate knowledge.
10	^77^Chatterjee, Pereira & Sarkar (2018)	Theory of Organizational Knowledge Creation	IT firms manager	Learning transfer system inventory foster organizational knowledge creation.
11	^78^Rusland, Jaafar & Sumintono (2020)	Theory of Organizational Knowledge Creation	Navy officers	Externalization and combination modes of knowledge conversion are more difficult to incorporate among the navy officers than socialization and internalization.
12	^79^Konno & Schillaci (2021)	Theory of Organizational Knowledge Creation	Entrepreneurs	Adding entrepreneurial activities to the SECI model as experimental processes.

### KC-IT project literature in three categories

KC-IT literature can be classified into three categories (see Tables 9 and 10) of the KC process, method and factor. The papers are presented in the following table by three categories as suggested by Mitchell and Boyle. ^20^ The benefit of viewing KC-IT literature in three categories include a better understanding of the current landscape of KC-IT.

**Table 9.** KC process.

Knowledge Creation (KC) Process
KC Process has 12 papers.
1. Kippenberger elevated organisational KC, which made information available and amplified it. ^68^
2. Eliufoo performed a case study looks at how construction firms can map and understand KC processes. ^69^
3. Virtual socialising mode in IT software businesses. ^70^
4. Matysiewicz *et al.* investigates the mechanisms of KC and how they affect network members. ^71^
5. A new Socialization-Externalization-Combination-Internalization (SECI) model was developed to explore how engineers generate and disseminate knowledge. ^72^
6. Marsina *et al.* found there Is lack of IT adoption in Slovakia enterprises. ^73^
7. Shongwe found a lack of software engineers may create knowledge from a number of sources, including lectures, older students, and professionals. ^74^
8. I-Ching and knowledge dynamics were combined by Yao, Han, and Li. ^75^
9. Moraes *et al.* discovered which aspects impact organisational socialisation and knowledge acquisition during innovation. ^76^
10. A theoretical framework built by Chatterjee, Pereira, and Sarkar was created using data from the SECI model and KC. ^77^
11. The Royal Malaysian Navy looks into its members’ comments to learn about present-day processes of KC in the fleet. ^78^
12. Konno and Schillaci introduced a paradigm linking knowledge generation to intellectual capital in society 5.0. ^79^

**Table 10.** KC method.

KC methods
This dimension consists twenty journals.
1. Mir and Rahaman observed that the workforce provides useful new information for the company. ^37^
2. Discourse Semantic Authoring (DSA) was suggested by Kamimaeda, Izumi, and Hasida as a technique to evaluate discussion participants’ contributions to knowledge development. ^38^
3. Inter-relational network foster knowledge creation. ^13^
4. Broadband internet technology is being utilised to distribute agricultural knowledge in Nigeria. ^39^
5. Knowledge creation categories include process, method and factor. ^20^
6. Wu *et al.* built a theoretical framework known as the Ontological SECI model. ^40^
7. Song, Uhm and Yoon surveyed measurement instruments for assessing organisational knowledge production. ^41^
8. Geo-referencing software helps explicit information become tacit. ^42^
9. Durst *et al.* discovered that networking activities foster knowledge creation. ^43^
10. Knowledge creation facilitates innovation capacity development. ^44^
11. Playfulness from event and dialogue facilitate knowledge creation. ^45^
12. Brix suggested that knowledge creation and organisational learning are integrated. ^46^
13. To learn about oneself and develop one’s knowledge, team skills and collaboration are critical for producing new knowledge. ^47^
14. Faccin and Balestrin built a theoretical framework to study factors of collaborative practise in R&D projects. ^34^
15. Li *et al.* suggested a novel knowledge production model integrating SECI with both explicit and tacit knowledge in high-technology projects. ^48^
16. Salehi *et al.* suggested conference and clinical unit for exchanging knowledge of clinical experiences. ^49^
17. Chin *et al.* established a new model (Polychronic KC) to help promote time as the new dimension in global IT industry. ^8^
18. Knowledge creation regardless of physical location. ^50^
19. Wang and Li applied statistical simulation using evolutionary game theory. ^51^
20. Digital gadgets assure the socio-psychological components of the learning process. ^52^

KC Factor: This dimension included 23 papers. We further classified the papers into three sub-dimensions of KC factors as suggested by Thani and Mirkamali ^32^ ( Table 11). Table 12 presents the summary of the 5 papers obtained when we have searched for the keyword combination of KC IT Project for DI. However, only 2 papers were found to have some relation to KC-TI-DI

**Table 11.** Summary of KC factors by the three types of factors.

Personal factor	Institutional factor	Support factor
• Goodwill, commitment, ethic of contribution, high care, atmosphere, wise leadership, love and friendship. ^28^ • Intention, autonomy, redundancy, variety. ^57^ • Basic skills of knowledge creation, motivation, time management, professional ethic, learning, teaching responsibility. ^32^ • Shared goal and hope. ^64^ • Creativity. ^66^ • TMS ^26^^,^^27^ and Trust. ^28^^-^^31^	• Knowledge network, graduate education, organization effectiveness. ^53^ • Organizational culture and social capital. ^54^ • Leadership, teamwork, corporate culture, and human resource management. ^55^ • Organizational communication, feedback promotion, policy formulation, information sharing. ^58^ • Organizational identity, mobility direction, human capital. ^56^ • Enabling structure, knowledge-creating culture, collaborative management, sabbatical, workforce development, interdisciplinary studies. ^32^ • Team safety and team learning. ^61^ • Talent management processes. ^62^ • Organic structure and organizational culture ^63^ and Information culture. ^65^	• Library, laboratory, infrastructure ^32^ and Social media. ^60^

**Table 12.** Summary of five papers on KC in IT projects for digital innovation.

Author	Theory used	Respondent group	Key findings
KC in IT Project for Digital Innovation (2 papers)			
^21^Ordieres-Meré *et al.* (2020)	Organization sustainability theory	Organization workers	Industry4.0 has a close relationship with the three elements of sustainability: economic, environmental and social sustainability. A relationships exists between knowledge creation and sustainability via Industry4.0 as the primary driver.
^22^Van den Berg (2019)	Teaching Innovation	Universities	Digital innovation skills including ‘meta-knowledge’ which refers to the information required to drive creativity, innovative, problem-solving, critically, communication, and collaboration.
^23^Park *et al.* (2015)	Knowledge creation process philosophy	Firms employees	A case study shows that the idea centre continues to evolve and members of production teams produce knowledge as a result of their activities and interactions.
^24^Kyakulumbye, Pather & Jantjies (2019)	Personal constructs theory, Situation awareness theory	Universities	User friendliness and relevance are critical knowledge structures for system assessment. System performance and interface attractiveness promote ease of use.
^25^Shimamoto (2011)			Japanese chemical companies’ R&D strategy changed from commercialization to diversification, and then transformed to specialized strategy.

**Table 13.** Summary of theories used in papers.

Theory	Count
Theory of Organizational Knowledge Creation (TOKC)	34
Knowledge creation capability, Transactive memory system	1
Organisational learning theory, The learning organisation, TOKC, Knowledge-integration theory, Communities of practice theory	1
Organizational justice theory, Relationship marketing theory	1
Resource-based view, TOKC	2
Social capital theory, Organizational culture theory	1
Organizational learning theory, TOKC	1
Concept of Ba, TOKC	1
Transactive memory system, Knowledge management theory, TOKC	1
Discourse semantic authoring theory	1
Evolutionary game theory	1
Information culture theory	1
Innovative organisations theory	1
Knowledge creation enablers theory	2
Knowledge-based view	1
Play theory	1
Systems model of creativity theory	1
Talent management processes theory	1
Team psychological safety theory	1
Paper without theory	3

**Figure 8.**
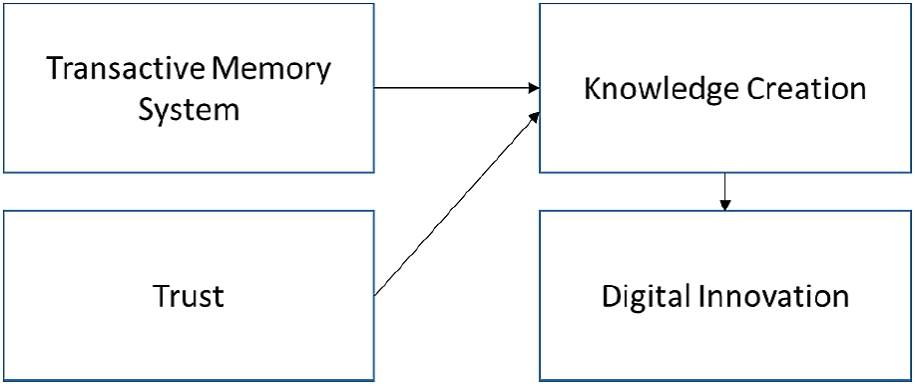
Proposed theoretical framework.

### Theories for KC-IT-DI

A total of 25 different theories were employed in the 57 papers analysed. 34 papers have used the TOKC by Nonaka and Takeuchi as the kernel theory. ^4^ The theories are listed in Table 13.

However, hardly any research mentioned TOKC in KC-IT-DI papers. Therefore, this scarcity is a research gap.

### Framework for KC-IT-DI

The proposed theoretical framework suggests that TMS and trust are important factors for influencing KC. KC will enable DI to create new products and services. The proposed framework was developed based on the findings in Table 7. Past literature indicated that transactive memory system (TMS) ^27^ and Trust ^28^^,^^31^^,^^33^ are positively related to KC. Hence, TMS are included in the framework.

### Limitations in current research and recommendation for future investigations

Limited research is available in KC in IT projects for DI. Past studies have not succinctly explained how knowledge may be applied to improve DI. Therefore, the KC-IT-DI literature is in its infancy and may warrant additional research. DI is important to the nation. ^34^ KC-IT offers additional benefits, including improving existing processes, introducing new business models and setting up new service channels. ^8^ To modernise products and services, KC-IT should be closely associated with DI. ^35^

Another limitation is the choice of keywords, which is determined by the study's emphasis. As a result, it is possible publishing bias. If the keywords are widened to cover non-specific fields of study, more articles may be acquired.

Present review suggested alternative theoretical underpinning such as investigate moderating effects relates to KC-IT-DI and factors that have underpinned existing research. ^36^ Future research should be carried out in the following areas:

1. More research focusing on KC-IT-DI will help researchers understand the significance of KC-IT in DI. Researchers may gain a better grasp of the issues afflicting the KC community.
2. TMS foster individuals to distribute and exchange tacit knowledge for their own advantage, as indicated by Dunaway and Sabherwal ^26^ and Çetin. ^27^ Therefore, exploring how TOKC plays its roles in TMS is recommended.
3. Examining new variables or dimensions in the KC-IT-DI relationship is a means of extrapolating novel aspects to boost KC and innovation in the IT industry in the context of volatility, uncertainty, complexity, and ambiguity.

## Conclusion

Three main points are addressed in this study. Firstly, the SLR found gaps in KC-IT linkage to DI. Secondly, TMS and trust are essential to KC. Finally, KC-IT-DI research limitations were addressed. This work advances the understanding of IT project management by studying the underlying factors to comprehend KC’s role in IT projects. This article mentions previous contributions other than the current concerns. This research focused on KC for interdisciplinary study. The implications herein provide relevant research and education references for researchers and the public. This work will also help scholars by offering directions. The shortcoming of the current study highlights the challenges in KC-IT-DI research. Furthermore, this article revealed a gap in KC in relation to IT projects, and the community is asked to research further to fill this gap.

## Data availability

### Figshare. Data File.xlsx

DOI: https://doi.org/10.6084/m9.figshare.14870655.v1

This project contains the following data:

This dataset is analysed for theories, type of papers, Knowledge Creation and Information Technology (KC-IT) factors, process, and method. ^80^

### PRISMA checklist

Figshare. PRISMA checklist 2020

DOI: https://doi.org/10.6084/m9.figshare.16692208.v1. ^81^

### PRISMA flowchart

Figshare. PRISMA checklist

DOI: https://doi.org/10.6084/m9.figshare.16657309.v1. ^82^

Data are available under the terms of the Creative Commons Attribution 4.0 International license (CC-BY 4.0).

## References

[1] Ramona T Alexandra B : Knowledge retention within small and medium sized enterprises. *Studies in Busin. Econom.* 2020;14(3):231–238. Scopus 10.2478/sbe-2019-0056

[2] Davenport TH Prusak L : *Working knowledge: How organizations manage what they know.* Harvard Business Press;1998.

[3] Bundesministerium für Wirtschaft und Energie (BmWi): Was ist Industrie 4.0? Menschen, Maschinen und Produkte sind direkt miteinander vernetzt: die vierte industrielle Revolution hat begonnen. 2020. Date of download: 1.6.2021. Reference Source

[4] Nonaka I Takeuchi H : *The knowledge-creating company: How Japanese companies create the dynamics of innovation.* Oxford University Press;1995.

[5] Argote L McEvily B Reagans R : Managing knowledge in organizations: an integrative framework and review of emerging themes. *Manag. Sci.* 2003;49:571–582. 10.1287/mnsc.49.4.571.14424

[6] Farnese ML Barbieri B Chirumbolo A : Managing knowledge in organizations: A Nonaka’s SECI model operationalization. *Front. Psychol.* 2019;10:2730. 10.3389/fpsyg.2019.02730 31920792 PMC6914727

[7] Foord D McLaughlin JD : Embedded research in energy innovation: An examination of cases and theories of knowledge creation for power generation and lighting technologies. *Energy Res. Soc. Sci.* 2019;56:101211. 10.1016/j.erss.2019.05.021

[8] Chin T Wang S Rowley C : Polychronic knowledge creation in cross-border business models: A sea-like heuristic metaphor. J. Knowl. Manag. 2020;25:1–22. (ahead-of-print). 10.1108/JKM-04-2020-0244

[9] Xiang Z Fesenmaier DR Werthner H : Knowledge creation in information technology and tourism: A critical reflection and an outlook for the future. *J. Travel. Res.* 2021;60(6):1371–1376. 10.1177/0047287520933669

[10] Wagner H Finkenzeller T Würth S : Individual and team performance in team-handball: A review. *J. Sports Sci. Med.* 2014;13(4):808. 25435773 PMC4234950

[11] Panahi S Watson J Partridge H : Conceptualising social media support for tacit knowledge sharing: physicians’ perspectives and experiences. *J. Knowl. Manag.* 2016. 10.1177/1460458214540907

[12] O’Riordan T : *The Transition to Sustainability: The Politics of Agenda 21 in Europe.* Routledge;2013. 10.4324/9781315071060

[13] Nambisan S Lyytinen K Majchrzak A : Digital innovation management: Reinventing innovation management research in a digital world. *MIS Q.* 2017;41:223–238. 10.25300/MISQ/2017/41:1.03

[14] Nasiri M Ukko J Saunila M : Managing the digital supply chain: The role of smart technologies. *Technovation.* 2020;96:102121. 10.1016/j.technovation.2020.102121

[15] Barnier AJ Klein L Harris CB : Transactive Memory in Small, Intimate Groups: More Than the Sum of Their Parts. *Small Group Res.* 2018;49(1):62–97. 10.1177/1046496417712439

[16] Pagano A Carloni E Galvani S : The dissemination mechanisms of Industry 4.0 knowledge in traditional industrial districts: evidence from Italy. *Competitiveness Review: An International Business Journal.* 2020;31(1):27–53. 10.1108/CR-12-2019-0160

[17] Holmström J : Recombination in digital innovation_ Challenges, opportunities, and the importance of a theoretical framework. *Inf. Organ.* 2018;4.

[18] Tranfield D Denyer D Smart P : Towards a Methodology for Developing Evidence-Informed Management Knowledge by Means of Systematic Review. *Br. J. Manag.* 2003;14(3):207–222. 10.1111/1467-8551.00375

[19] Moher D Liberati A Tetzlaff J : Preferred Reporting Items for Systematic Reviews and Meta-Analyses: The PRISMA Statement. *PLoS Med.* 2009;6(7):e1000097. 10.1371/journal.pmed.1000097 19621072 PMC2707599

[20] Mitchell R Boyle B : Knowledge creation measurement methods. *J. Knowl. Manag.* 2010;14(1):67–82. 10.1108/13673271011015570

[21] Ordieres-Meré J Prieto Remón T Rubio J : Digitalization: An Opportunity for Contributing to Sustainability From Knowledge Creation. *Sustain. For.* 2020;12(4):1460. 10.3390/su12041460

[22] Van den Berg C : Teaching Innovation to Strengthen Knowledge Creation in a Digital World. *Elect. J. Knowl. Manag.: EJKM.* 2019;17(2):144–157. 10.34190/EJKM.17.02.004

[23] Park HY Chang H Park Y-S : Firm’s knowledge creation structure for new product development. *Cogent Business & Manage.* 2015;2(1):1025307. 10.1080/23311975.2015.1023507

[24] Kyakulumbye S Pather S Jantjies M : Knowledge Creation in a Participatory Design Context: The use of Empathetic Participatory Design. 2019;49–65.

[25] Shimamoto M : R&D Strategy and Knowledge Creation in Japanese Chemical Firms (1980 – 2010). n.d.;15.

[26] Dunaway MM Sabherwal R : Understanding the role of Transactive Memory Systems and Knowledge Management Mechanisms on Team Performance. 2012. Reference Source

[27] Çetin S : The Effects of Transactive Memory Systems, Collective Mind and Innovative Culture on Knowledge Creation Capability. *Business & Management Studies: An International Journal.* 2019;7(1):563–578. 10.15295/bmij.v7i1.1092

[28] Castro G Sánchez Á : Exploring Knowledge Creation and Transfer in the Firm: Context and Leadership*/Explorando la creación y transferencia de Conocimiento en la empresa: Contexto y Liderazgo. *Univ. Bus. Rev.* 2013;40:126–137.

[29] Sankowska A : Relationships between organizational trust, knowledge transfer, knowledge creation, and firm’s innovativeness. *Learn. Organ.* 2013;20(1):85–100. 10.1108/09696471311288546

[30] Teerajetgul W Charoenngam C : Factors inducing knowledge creation: Empirical evidence from Thai construction projects. *Eng. Constr. Archit. Manag.* 2006;13(6):584–599. 10.1108/09699980610712382

[31] Tootell A Kyriazis E Billsberry J : Knowledge creation in complex inter-organizational arrangements: Understanding the barriers and enablers of university-industry knowledge creation in science-based cooperation. *J. Knowl. Manag.* 2020;25(4):743–769. 10.1108/JKM-06-2020-0461

[32] Thani FN Mirkamali SM : Factors that enable knowledge creation in higher education: A structural model. *Data Techn. Applic.* 2018;52(3):424–444. 10.1108/DTA-10-2016-0068

[33] Wang L Zhang M Li X : Trust and knowledge creation: The moderating effects of legal inadequacy. *Ind. Manag. Data Syst.* 2017;117(10):2194–2209. 10.1108/IMDS-11-2016-0482

[34] Faccin K Balestrin A : The dynamics of collaborative practices for knowledge creation in joint R&D projects. *J. Eng. Technol. Manag.* 2018;48:28–43. 10.1016/j.jengtecman.2018.04.001

[35] Goyal S Ahuja M Kankanhalli A : Does the source of external knowledge matter? Examining the role of customer co-creation and partner sourcing in knowledge creation and innovation. *Inf. Manag.* 2020;57(6):103325. 10.1016/j.im.2020.103325

[36] Paul J Lim WM O’Cass A : Scientific procedures and rationales for systematic literature reviews (SPAR-4-SLR). *Int. J. Consum. Stud.* 2021. 10.1111/ijcs.12695

[37] Mir M Rahaman AS : Organisational knowledge creation and the commercialisation of State mail service. *Int. J. Public Sect. Manag.* 2003;16(5):373–392. 10.1108/09513550310489313

[38] Kamimaeda N Izumi N Hasida K : Evaluation of participants’ contributions in knowledge creation based on semantic authoring. *Learn. Organ.* 2007;14(3):263–280. 10.1108/09696470710739426

[39] Ha L Nnajiofor Okigbo R Igboaka P : Knowledge creation and dissemination in sub-Saharan Africa. *Manag. Decis.* 2008;46(3):392–405. 10.1108/00251740810863852

[40] Wu Y Senoo D Magnier-Watanabe R : Diagnosis for organizational knowledge creation: An ontological shift SECI model. *J. Knowl. Manag.* 2010;14(6):791–810. 10.1108/13673271011084862

[41] Song JH Uhm D Won Yoon S : Organizational knowledge creation practice: Comprehensive and systematic processes for scale development. *Leadersh. Org. Dev. J.* 2011;32(3):243–259. 10.1108/01437731111123906

[42] Zurita G Baloian N : Mobile, Collaborative Situated Knowledge Creation for Urban Planning. *Sensors.* 2012;12(5):6218–6243. 10.3390/s120506218 22778639 PMC3386738

[43] Durst S Edvardsson IR Bruns G : Knowledge creation in small building and construction firms. *J. Innov. Manage.* 2013;1(1):125–142. 10.24840/2183-0606_001.001_0009

[44] Esterhuizen D Schutte CSL Toit ASA du : Knowledge creation processes as critical enablers for innovation. *Int. J. Inf. Manag.* 2012;32(4):354–364. 10.1016/j.ijinfomgt.2011.11.013

[45] Suorsa AR : Knowledge creation and play – a phenomenological approach. *J. Doc.* 2015;71(3):503–525. 10.1108/JD-11-2013-0152

[46] Brix J : Exploring knowledge creation processes as a source of organizational learning: A longitudinal case study of a public innovation project. *Scand. J. Manag.* 2017;33(2):113–127. 10.1016/j.scaman.2017.05.001

[47] Elsa G Edvardsson IR Link to external site, this link will open in a new window : Knowledge Management, Knowledge Creation, and Open Innovation in Icelandic SMEs. *SAGE Open.* 2018;8(4):215824401880732. 10.1177/2158244018807320

[48] Li M Liu H Zhou J : G-SECI model-based knowledge creation for CoPS innovation: The role of grey knowledge. *J. Knowl. Manag.* 2018;22(4):887–911. 10.1108/JKM-10-2016-0458

[49] Salehi K Kermanshahi S Mohammadi E : The Nurses’ Experiences of the Knowledge Creation Space in Clinical Setting: A Qualitative Study. *Biosci. Biotechnol. Res. Asia.* 2018;15(2):369–376 10.13005/bbra/2641

[50] Choi H Gu C : Geospatial Response for Innovation in the Wine Industry: Knowledge Creation through Institutional Mobility in China. *Agronomy.* 2020;10(4):495. 10.3390/agronomy10040495

[51] Wang D Li B : Behavioral Selection Strategies of Members of Enterprise Community of Practice—An Evolutionary Game Theory Approach to the Knowledge Creation Process. *IEEE Access.* 2020;8:153322–153333. 10.1109/ACCESS.2020.3018188

[52] Pokrovskaia N Leontyeva VL Ababkova MY : Digital Communication Tools and Knowledge Creation Processes for Enriched Intellectual Outcome—Experience of Short-Term E-Learning Courses during Pandemic. *Future Inter.* 2021;13(2):43. 10.3390/fi13020043

[53] Miyashita F : On the contribution of Knowledge Creation and Information Technology in the Organization by applying the undirected and directed independent graph. 2003;9.

[54] Siadat SA Hoveida R Abbaszadeh M : Knowledge creation in universities and some related factors. *J. Manag. Dev.* 2012;31(8):845–872. 10.1108/02621711211253286

[55] Thang NN Quang T Son NH : Knowledge Creation and Green Entrepreneurship: A Study of Two Vietnamese Green Firms. *Asian Academy of Management Journal.* 2013;18(2):129–151.

[56] Lee J Park NK Kim H : The effect of change in organizational identity on knowledge creation by mobile R&D workers in M&As. *J. Organ. Chang. Manag.* 2014;27(1):41–58. 10.1108/JOCM-12-2012-0195

[57] Begoña Lloria M Peris-Ortiz M : Knowledge creation. The ongoing search for strategic renewal. *Ind. Manag. Data Syst.* 2014;114(7):1022–1035. 10.1108/IMDS-01-2014-0011

[58] Nair AC Ramalingam S Ravi A : Knowledge Creation Within the Automobile Industry. *Intern. J. Eng. Business Manage.* 2015;7:16–16. 10.5772/61090

[59] Mikhaylov NS : Curiosity and its role in cross-cultural knowledge creation. *Int. J. Emot. Educ.* 2016;8(1):95–108.

[60] Papa A Santoro G Tirabeni L : Social media as tool for facilitating knowledge creation and innovation in small and medium enterprises. *Balt. J. Manag.* 2018;13(3):329–344. 10.1108/BJM-04-2017-0125

[61] Cauwelier P Ribiere VM Bennet A : The influence of team psychological safety on team knowledge creation: A study with French and American engineering teams. *J. Knowl. Manag.* 2019;23(6):1157–1175. 10.1108/JKM-07-2018-0420

[62] Mohammed AA Baig AH Gururajan R : The effect of talent management processes on knowledge creation: A case of Australian higher education. *J. Industry-University Collab.* 2019;1(3):132–152. 10.1108/JIUC-05-2019-0010

[63] Stojanović-Aleksić V Erić Nielsen J Bošković A : Organizational prerequisites for knowledge creation and sharing: Empirical evidence from Serbia. *J. Knowl. Manag.* 2019;23(8):1543–1565. 10.1108/JKM-05-2018-0286

[64] Goswami AK Agrawal RK : Explicating the influence of shared goals and hope on knowledge sharing and knowledge creation in an emerging economic context. *J. Knowl. Manag.* 2019;24(2):172–195. 10.1108/JKM-09-2018-0561

[65] Tajedini O Tandiseh A : An Investigation of the Relationship Between Information Culture Components and Knowledge Creation Among Top Iranian Researchers. *Libr. Philos. Pract.* 2020;1–24.

[66] Yoon SK Kim JH Park JE : Creativity and knowledge creation: The moderated mediating effect of perceived organizational support on psychological ownership. *Euro. J. Train. Develop.* 2020;44(6/7):743–760. 10.1108/EJTD-10-2019-0182

[67] Merx-Chermin M Nijhof WJ : Factors influencing knowledge creation and innovation in an organisation. *J. Eur. Ind. Train.* 2005;29(2):135–147. 10.1108/03090590510585091

[68] Kippenberger T : A new theory of knowledge creation. *Antidote.* 1997;2(2):14–15. 10.1108/EUM0000000006333

[69] Eliufoo H : Knowledge creation in construction organisations: A case approach. *Learn. Organ.* 2008;15(4):309–325. 10.1108/09696470810879565

[70] Spraggon M Bodolica V : Knowledge creation processes in small innovative hi-tech firms. *Manag. Res. News.* 2008;31(11):879–894. 10.1108/01409170810913060

[71] Matysiewicz J Smyczek S : Knowledge Creation in International Scientific Networks on Example of NetAware Intensive Programme. *Equilibrium.* 2013;8(4):107–122. 10.12775/EQUIL.2013.029

[72] Naicker K Govender KK Naidoo K : Knowledge creation and transfer amongst postgraduate students: Original research. *South African J. Inform. Manag.* 2014;16(1):1–8. 10.4102/sajim.v16i1.609

[73] Marsina S Hamranova A Okruhlica F : Knowledge Creation and Learning within the Building Project Orientation of Organizations. *Procedia Manufact.* 2015;3:723–730. Scopus 10.1016/j.promfg.2015.07.315

[74] Shongwe MM : Knowledge-creation in student software-development teams. *South African J. Inform. Manag.* 2015;17(1):1–8. 10.4102/sajim.v17i1.613

[75] Yao W Han X Li Y : Cross-organizational knowledge creation theory from the perspective of I-Ching: Case study in Chinese aerospace industry. *Chin. Manag. Stud.* 2015;9(4):528–552. 10.1108/CMS-07-2015-0162

[76] Moraes CRB de Woida LM Valentim MLP : Informational Socialization for Knowledge Creation in the Electrical and Electronics Sector. *Brazilian J. Inform. Sci.: Res. Trends.* 2016;10(3). 10.36311/1981-1640.2016.v10n3.06.p44 Reference Source.

[77] Chatterjee A Pereira A Sarkar B : Learning transfer system inventory (LTSI) and knowledge creation in organizations. *Learn. Organ.* 2018;25(5):305–319. 10.1108/TLO-06-2016-0039

[78] Rusland SL Jaafar NI Sumintono B : Evaluating knowledge creation processes in the Royal Malaysian Navy (RMN) fleet: Personnel conceptualization, participation and differences. *Cogent Business & Manage.* 2020;7(1). 10.1080/23311975.2020.1785106

[79] Konno N Schillaci CE : Intellectual capital in Society 5.0 by the lens of the knowledge creation theory. *J. Intellect. Cap.* 2021;22(3):478–505. 10.1108/JIC-02-2020-0060

[80] Soon Seng T Dorasamy M Razak R : Data File.xlsx. figshare. *Dataset.* 2021. 10.6084/m9.figshare.14870655.v1 PMC8667007 34950455

[81] Soon Seng T Dorasamy M Razak R : PRISMA Checklist 2020. figshare. *Figure.* 2021; 10.6084/m9.figshare.16692208.v1

[82] Soon Seng T Dorasamy M : PRISMA Checklist. figshare. *Dataset.* 2021. 10.6084/m9.figshare.16657309.v1

