# Supplementary material for: Knowledge creation in IT projects to accelerate digital innovation: two decade systematic literature review
Source: F1000Res. 2021 Dec 2;10:1040. Originally published 2021 Oct 12. [Version 3] doi: 10.12688/f1000research.70646.3 (PMC8667007; doi:10.12688/f1000research.70646.3)
Supplement: Peer review history

# Peer review: Reviewer response for version 3

**DOI:** 10.5256/f1000research.79903.r101812

**Published:** 2021-12-07

**Authors:** Mohammad Jabbari

**Affiliations:** 1 School of Information Systems, Queensland University of Technology, Brisbane, QLD, Australia

I would like to thank the authors for their effort in addressing my concerns in the current version of the manuscript. The paper reads better and has been improved; however, there are still a few points that need careful consideration. The paper has some good potentials and addressing the remaining issues will improve the quality and impact of the paper.

 In Table 2, column 6, the authors used “knowledge citation” as a keyword. I assume it is a typo. But if not, it needs to be discussed in the text.

 The way results are presented is still confusing:

- The abstract says: *“This work provides a systemic literature review (SLR) to examine the literature in KC-IT and its connection to DI. A SLR of 527 papers from 2001 to 2021 was performed across six online databases. The review encompasses quantitative and qualitative studies on KC-IT factors, processes and methods. Three major gaps were found in the SLR. Firstly, only 57 (0.23%) papers were found to examine the association between KC and IT projects.” -* **This indicates that there were 527 relevant papers for this study.**
- Then on page three, the last paragraph says: “ *A total of 57 papers were found relevant to this study*.” - **This indicates that the other 470 papers were not relevant.**
- The first sentence in the discussion section says: “ ***Only two papers,** written by Ordieres-Meré et al. ^21^ and Van den Berg, ^22^ were pertinent to KC in IT projects for DI*.” In the following lines, however, it says: “ *we **found that only 5 papers** somehow touch base about KC-IT-DI.*” - **There should be consistency in how you report the findings.**

I like the theoretical framework, but it is still not clear how the authors developed this framework. The authors claim that the framework has been developed based on the findings from Table 7. It says, *“Past literature indicated that transactive memory system (TMS) ^27^ and Trust ^28,31,33^ are positively related to KC. Hence, TMS are included in the framework.”*. However, results in Table 7 show a group of other independent factors, such as Strategic Alignment, Culture, Leadership, etc. The authors may need to explain how they selected TMS and Trust among these variables or how they clustered their results into these TMS and Trust variables.

 I also suggest the authors explain how these results conclude that knowledge creation may lead to digital innovation.

 I understand that authors may have limited space, but I suggest the authors rethink how they reported Tables 5 and 6 to create space and address the remaining issues.

Are the rationale for, and objectives of, the Systematic Review clearly stated?

Partly

Is the statistical analysis and its interpretation appropriate?

Not applicable

Are sufficient details of the methods and analysis provided to allow replication by others?

Partly

Are the conclusions drawn adequately supported by the results presented in the review?

No

Reviewer Expertise:

Systems analysis and design, digital innovation

I confirm that I have read this submission and believe that I have an appropriate level of expertise to confirm that it is of an acceptable scientific standard.

# Peer review: Reviewer response for version 2

**DOI:** 10.5256/f1000research.79472.r100712

**Published:** 2021-11-22

**Authors:** Ab Razak Che Hussin

**Affiliations:** 1 Azman Hashim International Business School, University of Technology Malaysia, Johor, Malaysia

This article has been improved based on the suggestions that have been given previously. The flow and content are clear and the language is easy to understand. This article is suitable for SLR papers and sufficient for indexing.

Are the rationale for, and objectives of, the Systematic Review clearly stated?

Yes

Is the statistical analysis and its interpretation appropriate?

Partly

Are sufficient details of the methods and analysis provided to allow replication by others?

Yes

Are the conclusions drawn adequately supported by the results presented in the review?

Partly

Reviewer Expertise:

IT adoption and digital business improvement.

I confirm that I have read this submission and believe that I have an appropriate level of expertise to confirm that it is of an acceptable scientific standard.

# Peer review: Reviewer response for version 1

**DOI:** 10.5256/f1000research.74248.r96845

**Published:** 2021-11-02

**Authors:** Mohammad Jabbari

**Affiliations:** 1 School of Information Systems, Queensland University of Technology, Brisbane, QLD, Australia

This study aims to investigate knowledge creation in information technology projects for digital innovation through a systematic literature review. The study identified three main research gaps and proposed a framework to fill the gap. While I think the study is a relevant study and can provide potential contributions, I see major issues in the study that needs to be addressed.

 First: a good SLR "should strive to identify thematic gaps and theoretical biases, propose some future research directions, including alternative theoretical underpinnings, and not just stop at the summarizing/synthesizing stage." (Rowe, 2014, pg. 250 ^1^). This study claims that they have proposed a theoretical framework (Figure 7) that could potentially suggest future research directions. However, it is not clear how the framework was developed based on the findings of the SLR. I suggest that the authors include a section and discuss their framework development based on the results. 

 Second: The studly lacks a strong background. For example, the background should clearly specify what the authors mean by KC-IT:

- Do they mean KC during the IT development lifecycle?
- How does it differ from documentation, such as technical or user documentation?
- How can KC happen in IT projects?
- Do the authors mean DI for future IT projects, or do they mean DI in general which may include DI for business innovation, DI for product innovation, etc?

In summary, the scope of the work should be clearly explained and justified. 

 Third: the results in Table 4 clearly show that the search results for KC, IT and DI is 5 papers. Then the authors conclude that only 5 papers "are relevant to KC in IT projects to accelerate DI". Are these the same 5 papers identified through the search process or did you do some other analysis? A brief descriptive summary of search results may not provide enough contribution. You may need to explain your tables and figures in a more theoretical way. 

 Fourth: this study only analyzed 57 papers. The way results are reported is confusing. The results should explain how the results are derived from 57 papers, not the 527 papers! The percentage reported in the abstract and in the text should be out of 57 studies.

Are the rationale for, and objectives of, the Systematic Review clearly stated?

Partly

Is the statistical analysis and its interpretation appropriate?

Not applicable

Are sufficient details of the methods and analysis provided to allow replication by others?

Partly

Are the conclusions drawn adequately supported by the results presented in the review?

No

Reviewer Expertise:

Information Systems, Systems Analysis and Design, Conceptual Modeling, Digital Innovation

I confirm that I have read this submission and believe that I have an appropriate level of expertise to confirm that it is of an acceptable scientific standard, however I have significant reservations, as outlined above.

## References

[1] : What literature review is not: diversity, boundaries and recommendations. *European Journal of Information Systems* .2014;23(3) : 10.1057/ejis.2014.7 241-255 10.1057/ejis.2014.7

# Response

**Published:** 2021-11-29

**Authors:** Soon Seng Tung

**Affiliations:** Multimedia University, Malaysia

We thank you for all the valuable comments. We have addressed the comments as below:

**Introduction**

 The study lacks a strong background. For example, the background should clearly specify what the authors mean by KC-IT:

- Do they mean KC during the IT development lifecycle?
- How does it differ from documentation, such as technical or user documentation?
- How can KC happen in IT projects?

Do the authors mean DI for future IT projects, or do they mean DI in general which may include DI for business innovation, DI for product innovation, etc?

**Response to Comments:**

 Thank you for these comments. We have included the following sentences in the text now.

- *KC-IT is referring to transfer of expertise and knowledge generated at different time in IT project. It is beyond the IT development lifecycle (Xiang et al., 2021).*
- *KC-IT does not limited to create documentations but it involves social media that overcomes the limitations in the traditional KC activities (Wagner et al., 2014, Panahi et al., 2016).*
- *For example, project team members create their project knowledge and expertise via social media platform.*
- *DI is essential for general business process and market offerings as technology evolves (Nasiri et al., 2020)*

**Result**

 In summary, the scope of the work should be clearly explained and justified. 

 Fourth: this study only analyzed 57 papers. The way results are reported is confusing. The results should explain how the results are derived from 57 papers, not the 527 papers! The percentage reported in the abstract and in the text should be out of 57 studies.

**Response to Comments:**

 Thank you for the comments. We have now rephrased it as follows:

*A total of 527 papers were identified by referring to the keyword search for KC-IT. 57 papers were found for the keyword search KC-IT-DI which belongs to subset of KC-IT.*

**Discussion**

- Third: the results in Table 4 clearly show that the search results for KC, IT and DI is 5 papers. Then the authors conclude that only 5 papers "are relevant to KC in IT projects to accelerate DI". Are these the same 5 papers identified through the search process or did you do some other analysis? A brief descriptive summary of search results may not provide enough contribution. You may need to explain your tables and figures in a more theoretical way. 
- This study claims that they have proposed a theoretical framework (Figure 7) that could potentially suggest future research directions. However, it is not clear how the framework was developed based on the findings of the SLR. I suggest that the authors include a section and discuss their framework development based on the results. 

**Response to Comments:**

- Thank you for the valuable comment. We have explained the result under the discussion section to relate to the theory.
- We have now inserted new headings and described the proposed framework which was developed based on the findings in Table 7. Past literature showed that transactive memory system (TMS) (Çetin, 2019) and Trust (Sankowska, 2013, Tootell, 2020, Wang *et al.*, 2020) are positively related to KC. Hence, we include TMS into the framework. **Recommendation for future investigations**

A good SLR "should strive to identify thematic gaps and theoretical biases, propose some future research directions, including alternative theoretical underpinnings, and not just stop at the summarizing/ synthesizing stage." (Rowe, 2014, pg. 250 ^1^ ).

**Response to Comments:**

 Thank you for these comments.

- We have added this line in the future investigation:

*‘Present review suggested alternative theoretical underpinning such as investigate moderating effects relates to KC-IT-DI and factors that have underpinned existing research. (Paul et al., 2021)’*

**References:**

1. Xiang, Z., Fesenmaier, D. R., & Werthner, H. (2021). Knowledge creation in information technology and tourism: A critical reflection and an outlook for the future. Journal of Travel Research, 60(6), 1371-1376.
2. Wagner, H., Finkenzeller, T., Würth, S., & Von Duvillard, S. P. (2014). Individual and team performance in team-handball: A review. Journal of sports science & medicine, 13(4), 808.
3. Panahi, S., Watson, J., & Partridge, H. (2016). Conceptualising social media support for tacit knowledge sharing: physicians’ perspectives and experiences. Journal of Knowledge Management.
4. Nasiri, M., Ukko, J., Saunila, M., & Rantala, T. (2020). Managing the digital supply chain: The role of smart technologies. Technovation, 96, 102121.
5. Çetin S: The Effects of Transactive Memory Systems, Collective Mind and Innovative Culture on Knowledge Creation Capability. Business & Management Studies: An International Journal. 2019;7(1):563–578. 10.15295/bmij.v7i1.1092
6. Sankowska A: Relationships between organizational trust, knowledge transfer, knowledge creation, and firm’s innovativeness. Learn. Organ. 2013;20(1):85–100. 10.1108/09696471311288546
7. Tootell A, Kyriazis E, Billsberry J, et al.: Knowledge creation in complex inter-organizational arrangements: Understanding the barriers and enablers of university-industry knowledge creation in science-based cooperation. J. Knowl. Manag. 2020;25(4):743–769. 10.1108/JKM-06-2020-0461
8. Wang D, Li B: Behavioral Selection Strategies of Members of Enterprise Community of Practice—An Evolutionary Game Theory Approach to the Knowledge Creation Process. IEEE Access. 2020;8:153322–153333. 10.1109/ACCESS.2020.3018188
9. Paul, J., Lim, W. M., O’Cass, A., Hao, A. W., & Bresciani, S. (2021). Scientific procedures and rationales for systematic literature reviews (SPAR‐4‐SLR). International Journal of Consumer Studies.

# Peer review: Reviewer response for version 1

**DOI:** 10.5256/f1000research.74248.r97965

**Published:** 2021-11-01

**Authors:** Ab Razak Che Hussin

**Affiliations:** 1 Azman Hashim International Business School, University of Technology Malaysia, Johor, Malaysia

**Introduction:**

- It may be necessary to explain a little more why KC is important in IT projects. After that, the relationship between IT and DI projects also needs to be properly explained for better understanding.
- There are differences between IT and IT projects and therefore please be sure to use them consistently in questions, objectives, and throughout the paper.

**Review Method:**

- The 6 databases covered in the SLR are good.
- Keywords, steps and extraction process are well executed.

**Result:**

- Further explanation is needed for each of Figures 5, 6, and 7 in terms of how they can be interpreted to the objectives of this study.
- Figure 8 may not be relevant here because it suddenly appears and there is no explanation about it. It may be moved at the end of the paper, or it may not be relevant in the SLR paper.

**Discussion:**

- The descriptions in the discussion should follow the sequence of SLR questions so that they are easy to understand.
- The key findings in each table can be taken from the table and explained in the paragraph after each table. This will improve the readability of the paper.

**Theory for KC-IT-DI:**

- It may be necessary to clarify KC-IT-DI requirements that do not exist in current SLRs.
- Figure 8 seems relevant to be placed here with further explanation of it.

**Limitations in current research and recommendations for future investigation:**

- The section title is not about limitations in current research. It should be a limitation of the previous study because the current study refers to the research conducted by the authors of this paper.

**Conclusion:**

- Good.

Are the rationale for, and objectives of, the Systematic Review clearly stated?

Yes

Is the statistical analysis and its interpretation appropriate?

Partly

Are sufficient details of the methods and analysis provided to allow replication by others?

Yes

Are the conclusions drawn adequately supported by the results presented in the review?

Partly

Reviewer Expertise:

IT adoption and digital business improvement.

I confirm that I have read this submission and believe that I have an appropriate level of expertise to confirm that it is of an acceptable scientific standard, however I have significant reservations, as outlined above.

# Response

**Published:** 2021-11-14

**Authors:** Soon Seng Tung

**Affiliations:** Multimedia University, Malaysia

Dear Dr Razak

 We thank you for all the valuable comments. We trust that the revised paper has addressed all the concerns. Thank you.

 Below are the author response to comments:

**Introduction**

- It may be necessary to explain a little more why KC is important in IT projects. After that, the relationship between IT and DI projects also needs to be properly explained for better understanding.
- There are differences between IT and IT projects and therefore please be sure to use them consistently in questions, objectives, and throughout the paper.

Author Response to Comments

 - Paragraphs in introduction is now improved.

 - Relationship between IT and DI projects has been explained.

 - Word ‘IT’ has been updated to ‘IT project’ accordingly.

**Review Method**

- The 6 databases covered in the SLR are good.
- Keywords, steps and extraction process are well executed.

Author Response to Comments

 - Thank you for these comments.

**Result**

- Further explanation is needed for each of Figures 5, 6, and 7 in terms of how they can be interpreted to the objectives of this study.
- Figure 8 may not be relevant here because it suddenly appears and there is no explanation about it. It may be moved at the end of the paper, or it may not be relevant in the SLR paper.

Author Response to Comments

 -Further explanation for Figures 5 and 7 is now added to indicate KC-IT research gaps.

 - Further explanation for Figure 6 depicted the objective of the study to understand the current view of the KC-IT literature in terms of sub categories.

 - Figure 8 placement was recommended by the editorial board hence no relocation was made.

**Discussion**

- The descriptions in the discussion should follow the sequence of SLR questions so that they are easy to understand.
- The key findings in each table can be taken from the table and explained in the paragraph after each table. This will improve the readability of the paper.

Author Response to Comments

 - Thank you for this comments. The descriptions in the discussion follows the sequence of SLR questions. - For example, it begins with answering the research gap in KC-IT in connection to DI. Next, the description highlighted TMS and trust affecting KC-IT. Third, it explains current view of KC-IT literature in terms of the KC process, method and factor. Lastly, discusses the underlying theories used by the literature.

 - Key findings in Table 4 and 5 are now improved.

**Theory of KC-IT DI**

- It may be necessary to clarify KC-IT-DI requirements that do not exist in current SLRs.
- Figure 8 seems relevant to be placed here with further explanation of it.

Author Response to Comments

 - The requirement for KC-IT-DI is the linkages between them. We have highlighted this in findings.

 - Explanation on Figure 8 is now provided.

**Limitations**

- The section title is not about limitations in current research. It should be a limitation of the previous study because the current study refers to the research conducted by the authors of this paper.

Author Response to Comments

 - Limitation of past studies are added.

**Conclusion**

- Good.

Author Response to Comments

 - Thank you for this comment.
